# Dr. Ferguson, on the Cure of Chancre

**Published:** 1803-12-01

**Authors:** T. Peaal


### *To the Editors of the Medical and Physical Journal.*

GENTLEMEN,

THE following letter I received some time since from Dr. Andrew Ferguson, of Aberdeen. I have tried the sulphat of soda poultice, which he recommends, in four cases of Chancre with the same success. That it might benefit others, I requested his liberty to transmit the letter I received from him to the Editors of the Medical and Physical Journal; with which he readily complied.

T. PEAAL.

---

"To Mr. T. PEAAL.

"SIR,

"Two years ago I had a Case of Syphilis, which appeared in a large chancre behind the glans penis under the prepuce. Before applying to me, he had been directed to use strong mercurial ointment, which he did without any good effect, the sore still continuing to have a foul and cancerous appearance. I ordered it to be dressed with an ointment prepared in the following manner.

℞. Ungt. hydrarg. fort. ungt. resin flav. aa ℥j. calomel ʒj. M. And a little of the hydrargyrus nitratus ruber to be sprinkled upon the surface of the sore.

Notwithstanding this had a fair trial, the chancre continued not only to look unfavourable but to increase in size. I changed the ointment. It was dressed with the following. ℞. Cerat. lapid. calaminar. ℥j. ceruss. acetat. hydrarg. nitrat. rub. aa gr. x. M. But with no better success. I considered, that ointment in this case was not likely to succeed, either in cleaning the sore or arresting its corroding nature. I then ordered him to apply a poultice to the chancre, prepared in the following manner. One ounce of the sulphat of soda was dissolved in 4 ℔. of boiling water. When perfectly cold, a little of this water was mixed with a small piece of the inner soft part of a loaf of wheaten bread, prepared of the finest flour; this was kneaded with the fingers until reduced to a soft poultice. It was then applied to the chancre, cold, and renewed six times every day. In the course of two days I could perceive a material difference in the appearance of the sore; and the patient said, that the pain was considerably abated. In ten days the whole surface of the chancre was red,

 new, new granulations of flesh springing up, and the discharge from it of a better quality, and much diminished in quantity. I used the same application until the chancre was completely whole, which happened in about one month after the first application of the sulphat of soda poultice. I looked upon this as a fortunate discovery, for I had frequently found a considerable difficulty in healing these venereal ulcers, especially when they were of a very inveterate and corroding kind. Upon making trial of this, I hope you will find the same success, for it is not in one solitary case that I have tried it, but in more than twelve, with always the same advantage. I must however observe, that when I have any suspicion of the venereal poison being absorbed, I never trust the cure to the poultice alone; I always cause my patients either to rub in strong mercurial ointment, or take mercurial pills until their mouths are affected by the mercury. The sulphat of soda poultice has a very great influence in preventing the absorption of the poison of syphilis; and in this, a great part of its virtue consists. For as the bread is of a mucilaginous nature, it mixes with the matter, and absorbs it, and this as soon as it is formed, which ointment never does, but confines it in a body upon the surface of the ulcer in as acrid a state as when it is secreted. Hence, one great advantage of this application is, that buboes are less readily formed. The sulphat of soda poultice, no doubt, produces a change in the action of the part, besides its absorbing the acrid matter. With this view, I tried it in two cases of herpes with the same good effect as in chancre. The quantity of sulphat of soda used in making the solution for the poultice was in these two cases much increased. Considering the powerful effect of the sulphat of soda in syphilitic ulcer, I thought it might also be a very good application in gonorrhœa. I tried it in six cases; it was dissolved in hot water; but did not answer so well as when used in the same manner as for chancre. The solution of sulphat of soda was mixed with wheaten bread, and dissolved to a thin mucilage; and, in this latter form, I will venture to say, that it is among the first injections that have been discovered.

"I remain, your's, &c.

"ANDREW FERGUSON."

OBSERVATIONS

